# Supplementary material for: Chromothripsis during telomere crisis is independent of NHEJ, and consistent with a replicative origin
Source: Genome Res. 2019 May;29(5):737–49. doi: 10.1101/gr.240705.118 (PMC6499312; doi:10.1101/gr.240705.118)
Supplement: Supplemental Material [file supp_gr.240705.118_Supplemental_file_1.zip › contigs/annotated_contigs/DB54/contig.2.DB54_length_326_mean_cov_4.75766871166.docx]

**DB54_length_326_mean_cov_4.75766871166**

```
CAAAGAATTGTCCTGTTTCATCTAGATTAGCAAATTTGTGGACAAAGAGTTCTTCATAATATTTCTTTACTATCTTCTTAATGTCCATG
 >chr9:105244397-105244603 - E=9e-110 p=0e+00
GGATCAGTAGAAATATGATGTTTGTGTGTGTGTCTGACAGGAATAATTTTTGTCATCACTGGTATTTTTCTACATTAGCTTGGCTAGAG

CTTTATCGCGTTCACTGATTTTTTTTTT|TATTGTCTCGTTTTGTTTTTTTTTGCTTTTTTTGTTG|TTTTGTTTATTTTGTTTTTTTG
                                                                  >chr7:117141801-11714
ATTTTTGATTTATTTTTTTTTTTTTTTCTTTTTTTTGTTTTTTTTTTTTTTT|TTTTTTTTT1874 - E=3e-10
```
